# Negative BOLD responses during hand and foot movements: An fMRI study

**DOI:** 10.1371/journal.pone.0215736

**Published:** 2019-04-19

**Authors:** Hiroki Nakata, Ryo Domoto, Nobuaki Mizuguchi, Kiwako Sakamoto, Kazuyuki Kanosue

**Affiliations:** 1 Faculty of Sport Sciences, Waseda University, Tokorozawa, Japan; 2 Health Sciences, Faculty of Human Life and Environment, Nara Women’s University, Nara City, Japan; 3 School of Sport Sciences, Waseda University, Tokorozawa, Japan; 4 The Japan Society for the Promotion of Science, Tokyo, Japan; 5 Faculty of Science and Technology, Keio University, Yokohama, Japan; 6 Department of Integrative Physiology, National Institute for Physiological Sciences, Okazaki, Japan; LUNEX International University of Health, Exercise and Sports, LUXEMBOURG

## Abstract

The present study employed functional magnetic resonance imaging (fMRI) to examine the characteristics of negative blood oxygen level-dependent (Negative BOLD) signals during motor execution. Subjects repeated extension and flexion of one of the following: the right hand, left hand, right ankle, or left ankle. Negative BOLD responses during hand movements were observed in the ipsilateral hemisphere of the hand primary sensorimotor area (SMI), medial frontal gyrus (MeFG), middle frontal gyrus (MFG), and superior frontal gyrus (SFG). Negative BOLD responses during foot movements were also noted in the bilateral hand SMI, MeFG, MFG, SFG, inferior frontal gyrus, middle temporal gyrus, parahippocampal gyrus, anterior cingulate cortex, cingulate gyrus (CG), fusiform gyrus, and precuneus. A conjunction analysis showed that portions of the MeFG and CG involving similar regions to those of the default mode network were commonly deactivated during voluntary movements of the right/left hand or foot. The present results suggest that three mechanisms are involved in the Negative BOLD responses observed during voluntary movements: (1) transcallosal inhibition from the contralateral to ipsilateral hemisphere in the SMI, (2) the deactivated neural network with several brain regions, and (3) the default mode network in the MeFG and CG.

## Introduction

Recent neuroimaging studies using functional magnetic resonance imaging (fMRI) reported not only increases, but also decreases in blood oxygen level-dependent (BOLD) signals during tasks. These decreases are often referred to as ‘Negative BOLD responses’, and several phenomena have been suggested to be involved. The first involves transcallosal inhibition from one hemisphere to the other. BOLD signals generally increase in the primary motor area (MI) of the contralateral hemisphere during the voluntary movement of a limb, but decrease in the MI ipsilateral to the movement [1–8]. The second phenomenon involves the task-related deactivation of associated areas that belong to an irrelevant sensory modality. For example, deactivation of the visual cortex occurs during somatosensory (tactile) discrimination tasks [9–12]. The third involves the “blood steal” phenomenon. When BOLD signals in some parts of the primary visual cortex increase after particular types of visual stimuli, signals in other parts of the visual cortex decrease [13, 14]. This is often explained by the blood steal phenomenon, which occurs due a decrease in blood flow (i.e. Negative response) in regions that are adjacent to activated regions with increased blood flow (i.e. Positive response) and supplied by a common artery. However, if the distance between Negative and Positive BOLD foci is large (e.g., left and right hemispheres, and frontal and occipital cortices), it is difficult to explain the relationship between Negative and Positive BOLD responses by the blood steal phenomenon [15]. The fourth is related to default mode network. This phenomenon comprises task-independent deactivation regions during the baseline or resting state of the brain involving a specific set of mental operations [16–18].

The physiological basis of Negative BOLD responses remains a matter of debate. Non-human studies revealed a relationship between Negative BOLD signals and decreases in neural activity [19–21]. Devor and colleagues [20] utilized a somatosensory stimulation in the rat to investigate neurovascular coupling in the primary somatosensory cortex (SI). They demonstrated that neuronal inhibition and concurrent arteriolar vasoconstriction were coupled with decreases in blood oxygenation, and that this may form the physiological basis for the Negative BOLD responses observed in the fMRI of humans. On the other hand, Maggioni and colleagues [22] reported a relationship between the neuronal rhythms at 10 and 12 Hz on electroencephalography and Negative BOLD responses in the extra-striate visual cortex, suggesting that Negative BOLD responses to visual stimuli were neuronal in origin rather than reflecting pure vascular phenomena.

As described above, previous studies reported Positive BOLD responses in the MI contralateral to the moved hand and Negative BOLD responses in the ipsilateral MI. Zeharia and colleagues [7] showed somatotopic organization in the MI for Negative BOLD responses. They demonstrated that the Negative BOLD spatial pattern in the MI was not randomly distributed, but was organized somatotopically across the entire MI. Negative BOLD responses were located outside the somatotopic location of the classified body parts (e.g., Negative BOLD elicited by bilateral hand movements were located in the leg and face areas). However, this study did not focus on Negative BOLD responses in brain regions other than the MI, and the underlying mechanisms of the Negative BOLD response across the entire brain during voluntary movement remain unclear. Furthermore, previous studies utilizing Positive BOLD responses reported neural substrates related to simple hand, foot, and tongue movements, and complex movements with the hand and foot [23, 24]. However, the laterality of the Negative BOLD response during even simple hand and foot movements has not yet been clarified. The objective of the present study was to elucidate how Negative BOLD responses are recruited across the whole brain during voluntary unilateral movements of the hands or feet on either side of the body.

The present study also used the term ‘primary somatosensory-motor area (SMI)’ to refer to a condition in which neural activities in the MI and/or SI were observed during voluntary movements. This is because corticocortical connections have been reported between the MI and SI [25], the distance between them is small and activation in the MI and SI may not be discriminated. Moreover, voluntary hand and foot movements themselves give rise to proprioceptive inputs. Therefore, many fMRI studies have used the term “SMI” to refer to condition in which voluntary movements were performed [26, 27]. We employed this usage of SMI in the present study.

## Materials and methods

### Subjects

Fifteen normal right-handed subjects (two females and thirteen males; mean age 20.8 years, range 18–25 years) participated in the present study. All subjects were undergraduate or graduate students. They were all right handed according to the criteria of the Edinburgh Inventory [28]. Subjects had no record of neurological or psychiatric disorders. The protocol was approved by the Human Research Ethics Committee of Waseda University, Japan. Subjects were informed in detail about the experiments prior to their participation, and gave written informed consent for the involvement in this study.

### Procedure

Recordings were conducted under four conditions: (1) right hand movement (RH), (2) left hand movement (LH), (3) right foot movement (RF), and (4) left foot movement (LF). Under the RH and LH conditions, subjects were asked to repeatedly perform extension and flexion of the right or left hand. Under the RF and LF conditions, subjects were instructed to repeatedly perform plantar flexion and dorsiflexion of the right or left ankle. Subjects were told to perform each movement at their own pace, and to not count the number of movements performed. In addition, they were asked to match the pace of the hand and foot movements throughout all of the experiments. A practice session of several trials for each movement was performed before the recording in order to enable subjects to become familiar with the experimental conditions.

Two experimenters monitored the number of movements under all of the conditions tested (S1 Table), and we performed an analysis of variance (ANOVA) with repeated measures using within-subject factors, limb (hand vs. foot), laterality (left vs. right), and block (1^st^, 2^nd^, 3^rd^, 4^th^, and 5^th^), to assess differences in motor performance between the various conditions. This analysis revealed that there was a main effect of limb (F (1, 14) = 6.028, p < 0.05), which indicated a significant difference in the speed of movement between the hand and foot conditions. Mean movement frequencies across all subjects were 1.33 Hz in the RH condition, 1.33 Hz in the LH condition, 1.16 Hz in the RF condition, and 1.14 Hz in the LF condition.

### fMRI data acquisition and analysis

On MRI, a 5-min 12-s run for each condition consisted of five alternate repetitions of the task and rest periods, each of which was 30 s. The first four volumes (12 s) of each fMRI session were discarded because of unstable magnetization. Subjects were informed about the start and end of each task period via a tactile cue. The cue was presented by an experimenter to the dorsum of the hand or foot that was to be subsequently moved or stopped from moving. The order of the four conditions (RH, LH, RF, and LF) was randomized for each subject and counterbalanced across all subjects. Subjects were also asked to keep their muscles relaxed and not to think about anything throughout the entire procedure. Any communication between the experimenter and subject was made via an intercom.

All images were acquired using a 1.5 T MR scanner (Signa, General Electric, Wisc., USA). BOLD contrast functional images were acquired using T2*-weighted echo planar imaging (EPI) free induction decay (FID) sequences with the following parameters: TR 3000 ms, TE 50 ms, FOV 22 × 22 cm, matrix size 64 × 64, flip angle 90°, slice thickness 5 mm, gap 1 mm, and 25 slices. The orientation of axial slices was parallel to the AC—PC line. T1-weighted images (TR 30 ms, TE 6 ms, FOV 24 × 24 cm, matrix size 256 × 256, flip angle 90°, slice thickness 1 mm, no gap, and 124 slices) were also obtained for each subject as an anatomical reference. These procedures generally followed those of previous studies [29, 30].

Raw data were analyzed using Statistical Parametric Mapping (SPM12, Wellcome Department of Cognitive Neurology, London, UK) [31] implemented in Matlab (Mathworks, Sherborn, Massachusetts, USA). The effects of head motion were corrected by realigning all scans to the first scan. Realigned images were normalized to the standard template of the Montreal Neurological Institute (MNI) brain using a transformation matrix obtained from the normalization process of the high-resolution image of each individual participant to the MNI template [32]. Images were then spatially smoothed using an isotropic Gaussian kernel of 8-mm full width at half maximum (FWHM) in the x, y, and z axes. Statistical analyses were performed on two levels. A first-level analysis was performed for each subject using a general linear model. Individual task-related activation and deactivation were initially evaluated. We constructed a statistical parametric map of the *t*-statistic for the eight contrasts: (1) Positive BOLD for RH, (2) Negative BOLD for RH, (3) Positive BOLD for LH, (4) Negative BOLD for LH, (5) Positive BOLD for RF, (6) Negative BOLD for RF, (7) Positive BOLD for LF, and (8) Negative BOLD for LF. Subject-specific contrast images of the estimated parameter were used for the second-level analysis (random-effect model) [33]. The second-level analysis utilizing a full factorial design (a one-way ANOVA, factor = limb, four levels) was performed to extend the inference of individual activation data to the general population. One-sample *t*-tests were used with a voxel-wise threshold of p < 0.0001 (uncorrected) to generate cluster images (spatial extent > 10 voxels). In addition, we evaluated the strong deactivation regions using the familywise error rate (FWE) with a cluster-level threshold of p < 0.05 for Negative BOLD responses for RH, LH, RF, and LF. We also checked whether the surviving cluster at the cluster-FWE threshold of p < 0.05 involved less than 10 voxels, but no such cluster was detected. The locations of brain activity were transformed from MNI coordinates into Talairach standard brain coordinates using the Talairach Daemon atlas [34]. This atlas was also used to localize the local maxima of the clusters in order to identify peak deactivation (activation) loci. All the coordinates were reported in the Talairach space. A conjunction analysis, to assess whether tasks altered activity in the same region of the brain, was also employed to detect brain regions commonly deactivated during all four (RH, LH, RF, and LF) conditions by utilizing SPM [35]. Using the SPM template, data were superimposed on a 3D-rendered standard brain. ‘Slightly in brighten blobs’ was selected as the ‘Display’ option for rendering in SPM [36–39]. We also confirmed the time course of the hemodynamic response (HDR) in each individual. Hand SMIs in the left and right hemispheres were selected based on Table 1. Each set of data was collected from all subjects using the ‘Plot’ option of SPM.

**Table 1:** Deactivation regions for each condition.

			Talairach coordinates			Z-score
Region	Side	BA	X	Y	Z	
<RH condition>						
Frontal Lobe						
Superior Frontal Gyrus	L	8	-20	26	47	4.60 #
	L	9	-8	51	20	4.11 #
Medial Frontal Gyrus	L	9	-16	40	22	4.57
	L	10	-2	53	12	4.74 #
	R	10	2	49	12	4.67
Middle Frontal Gyrus	L	8	-24	35	37	4.37
	L	9	-30	17	32	4.41 #
Precentral Gyrus	R	4	40	-19	53	4.40 #
	R	6	50	-5	50	4.08
Parietal Lobe						
Precuneus	L	7	-18	-53	38	4.04
Postcentral Gyrus	R	5	32	-44	57	4.07
Temporal Lobe						
Superior Temporal Gyrus	R	39	44	-51	27	4.40
Limbic Lobe						
Parahippocampal Gyrus	L		-28	-28	-7	4.55
	R	36	36	-34	12	4.01
Sub-lobar						
Insula	L	13	-42	26	8	4.06
	R	13	40	-9	19	4.44
Cerebellum (Tuber)	R		40	-65	-27	4.05
<LH condition>						
Frontal Lobe						
Superior Frontal Gyrus	R	11	12	48	-12	4.41
Medial Frontal Gyrus	L	6	-12	-26	60	3.88
	R	11	6	48	-11	4.18
Middle Frontal Gyrus	L	9	-28	33	35	4.82
Precentral Gyrus	L	4	-46	-13	54	4.23
Paracentral Lobule	L	6	-10	-32	55	3.97
	R	5	12	-32	50	4.38
Sub-lobar						
Insula	L	13	-32	-19	18	4.18
<RF condition>						
Frontal Lobe						
Superior Frontal Gyrus	L	8	-20	22	49	4.29
	R	9	26	35	31	4.90 #
Medial Frontal Gyrus	L	10	-8	42	-9	4.15 #
	R	10	4	49	9	4.07
Middle Frontal Gyrus	L	11	-30	36	-19	4.17
Precentral Gyrus	L	4	-40	-20	58	5.23 #
	R	4	36	-26	55	4.33 #
	R	6	30	-13	54	4.27
Inferior Frontal Gyrus	L	47	-24	15	-19	4.80
	R	13	34	13	-12	4.27 #
Parietal Lobe						
Postcentral Gyrus	L	3	-46	-17	52	4.93 #
	R	3	42	-17	52	4.11 #
Temporal Lobe						
Inferior Frontal Gyrus	R	13	32	5	-10	3.76
Middle Temporal Gyrus	R	21	50	-14	-13	4.09
Occipital Lobe						
Fusiform Gyrus	L	37	-34	-43	-11	4.76 #
Limbic Lobe						
Parahippocampal Gyrus	L	36	-24	-32	-14	5.23 #
	R	36	34	-35	-10	4.98 #
Anterior Cingulate	L	10	-12	50	-1	4.25 #
	L	24	-8	31	0	4.78 #
	L	32	-4	41	-2	3.83
	R	32	2	41	-2	3.99
Cingulate Gyrus	L	31	-10	-39	30	4.37 #
	R	31	8	-41	35	4.40 #
<LF condition>						
Frontal Lobe						
Middle Frontal Gyrus	R	9	26	37	35	4.42
Precentral Gyrus	L	4	-42	-17	41	3.98 #
	R	4	32	-18	38	4.46 #
	R	6	40	-12	32	3.97 #
Parietal Lobe						
Postcentral Gyrus	L	3	-42	-19	53	4.96 #
	R	3	42	-17	51	4.89 #
Precuneus	L	7	-16	-42	46	4.54
	R	7	4	-48	45	4.85 #
Temporal Lobe						
Superior Temporal Gyrus	R	22	51	-12	-1	4.10 #
Middle Temporal Gyrus	R	21	51	-12	-13	4.63 #
Sub-Gyral	R	21	48	-16	-13	4.55
Limbic Lobe						
Parahippocampal Gyrus	L	36	-24	-39	-6	3.75
	R		32	-6	-13	4.84 #
Cingulate Gyrus	L	31	-10	-35	33	4.19 #
Sub-lobar						
Lentiform Nucleus	L		-28	-18	-6	3.96

BA, Brodmann’s area; L, left hemisphere; R, right hemisphere

#, activation regions using the FWE with a cluster-level threshold of p < 0.05.

After the whole-brain analysis was completed, we conducted a region of interest (ROI) analysis to compare the strength of the hand SMI regions under each condition using MarsBaR (http://marsbar.sourceforge.net/). The coordinates of the ROI centers were defined by the local maximum voxel for the hand SMI in the group analysis. We detected the mean amplitude values of ROIs over the task block for each subject. Each ROI was defined as a 10-mm-radius sphere with the peak coordinates of the cluster (threshold p < 0.05, uncorrected), following a previous study using ROIs for SMIs [8]. The mean amplitude values of hand SMIs ipsilateral to the moving hand over the task block under the RH and LH conditions were evaluated by a repeated measures ANOVA using the factor of Condition (RH vs. LH). The mean amplitude values of bilateral hand SMIs over the task block under the RF and LF conditions were assessed by a repeated measures ANOVA using the factors of Condition (RF vs. LF) and Hemisphere (right vs. left). Significance was set at p < 0.05.

## Results

### Negative BOLD under each condition

**Fig 1:**
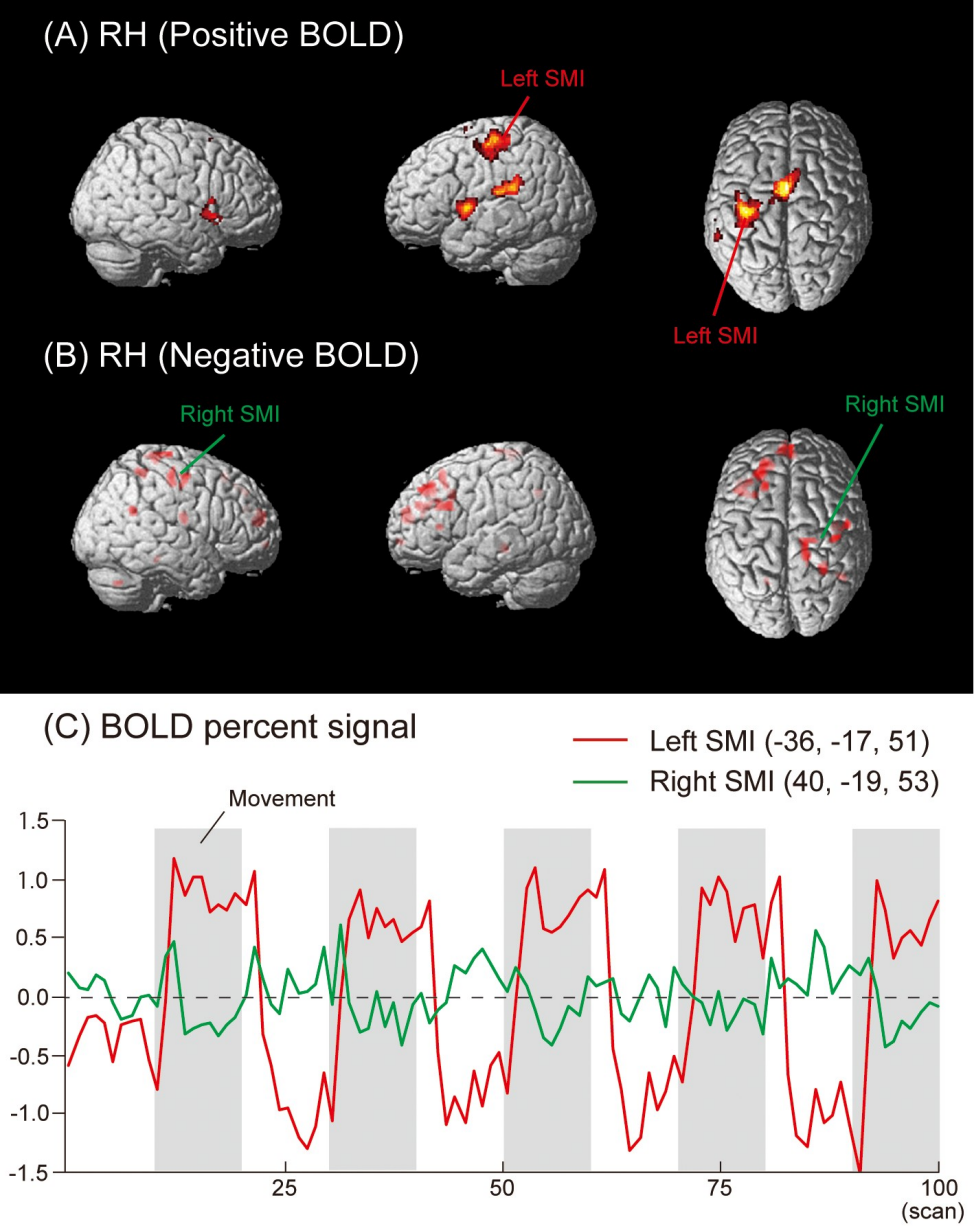
Group activation map showing (A) activated (Positive BOLD) and (B) deactivated (Negative BOLD) brain regions in the RH condition. (C) The mean time course of HDRs in the left (Positive BOLD) and right (Negative BOLD) SMI across all subjects. SMI = primary somatosensory-motor area.

Regions deactivated under the RH condition were located in the left superior frontal gyrus (SFG) (Brodmann’s areas: BA 8 and 9), medial frontal gyrus (MeFG) (BA 9 and 10), middle frontal gyrus (MFG) (BA 8 and 9), precuneus (BA 7), parahippocampal gyrus (PHG), and insula. Areas deactivated in the right hemisphere were in the SMI (BA 3/4), MeFG (BA 10), precentral gyrus (BA 6), postcentral gyrus (BA 5), superior temporal gyrus (STG) (BA 39), PHG (BA 36), insula (BA 13), and cerebellum (Tuber) (Fig 1, Table 1). The time courses of HDRs in the left (Positive BOLD) and right (Negative BOLD) SMI are shown in Fig 1C.

**Fig 2:**
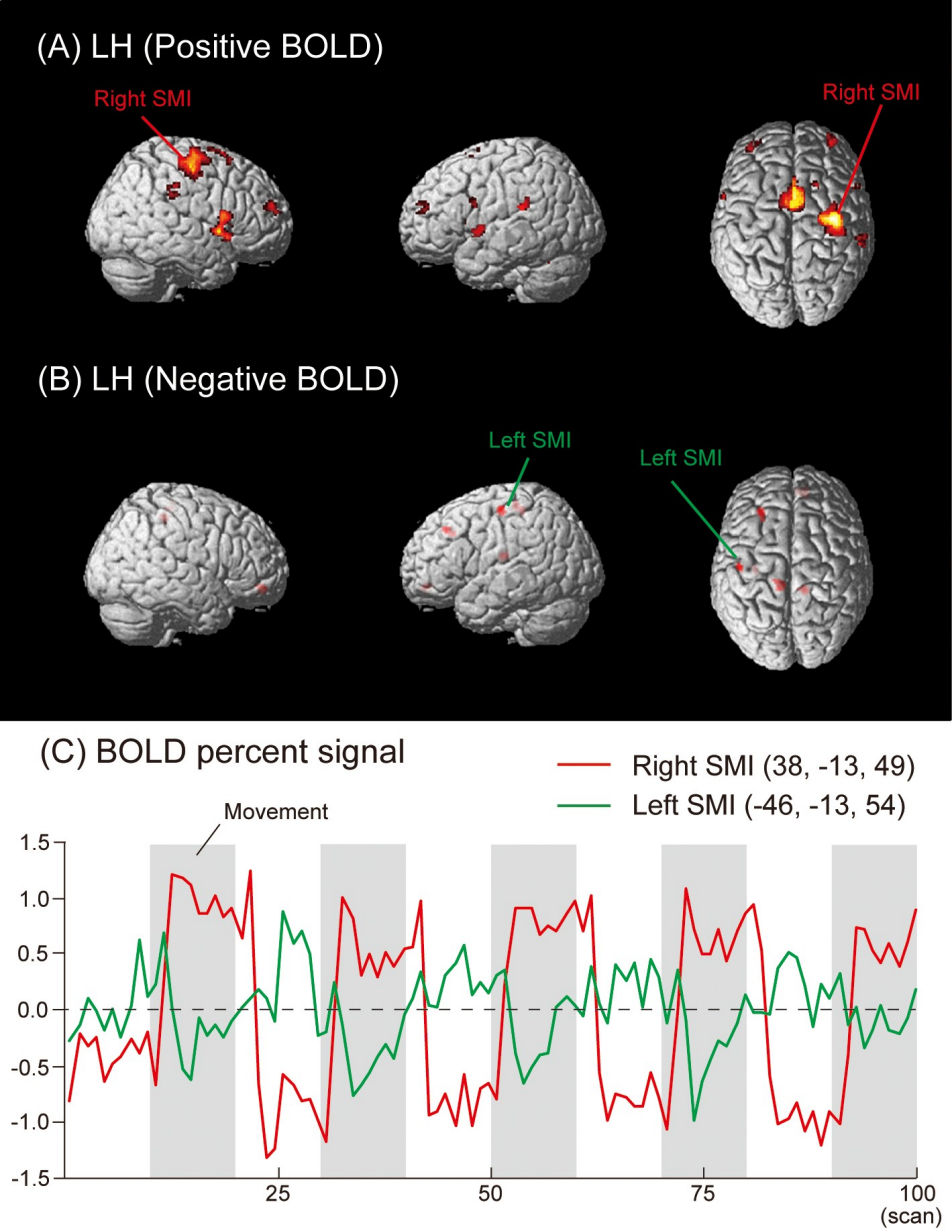
Group activation map showing (A) activated (Positive BOLD) and (B) deactivated (Negative BOLD) brain regions in the LH condition. (C) The mean time courses of HDRs in the right (Positive BOLD) and left (Negative BOLD) SMI across all subjects.

Regions deactivated under the LH condition were observed in the left SMI (BA 3/4), MeFG (BA 6), MFG (BA 9), paracentral lobule (BA 6), and insula (BA 13). Areas deactivated in the right hemisphere were in the SFG (BA 11), MeFG (BA 11), and paracentral lobule (BA 6) (Fig 2, Table 1). The time courses of HDRs in the right (Positive BOLD) and left (Negative BOLD) SMI are shown in Fig 2C.

**Fig 3:**
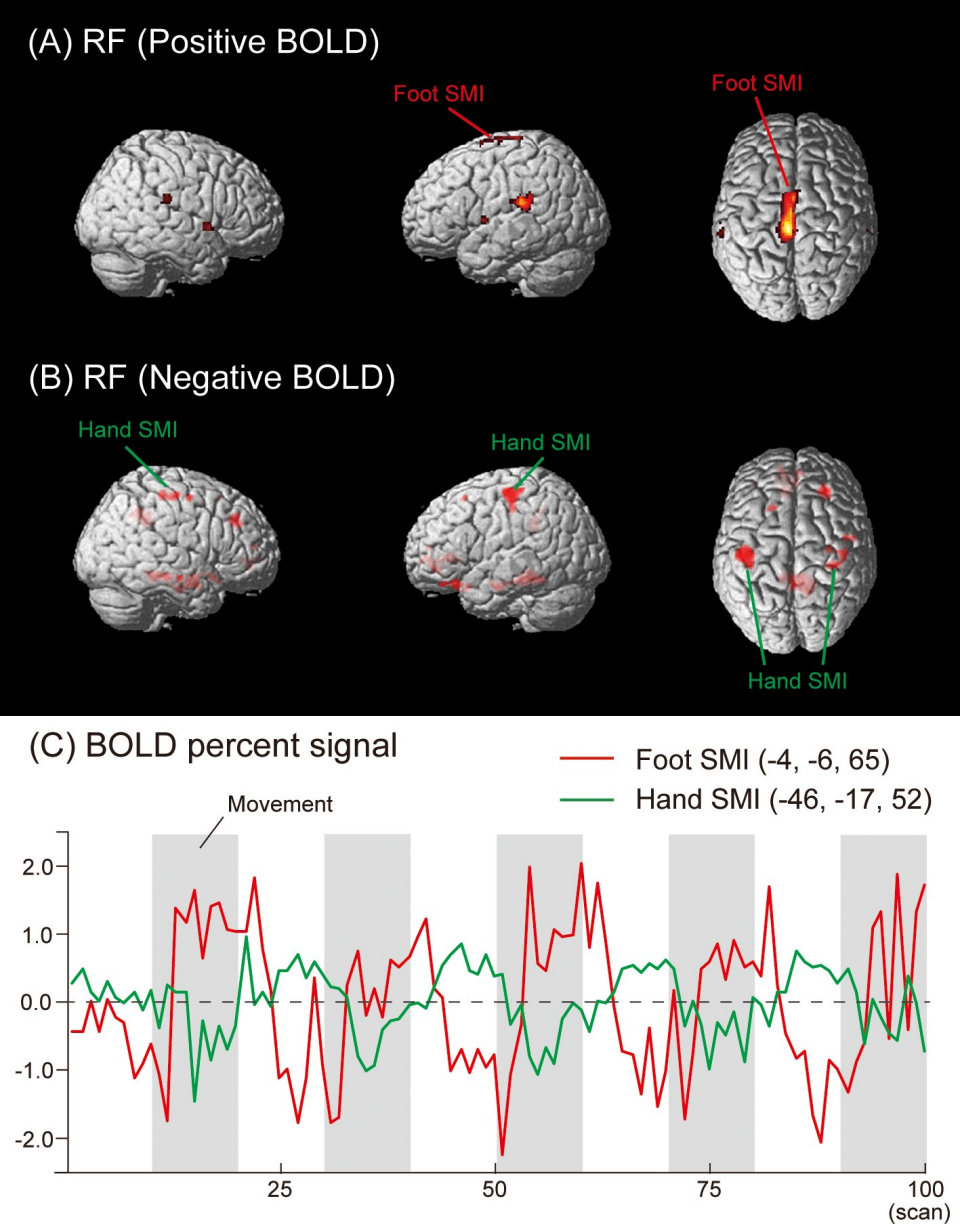
Group activation map showing (A) activated (Positive BOLD) and (B) deactivated (Negative BOLD) brain regions in the RF condition. (C) The mean time courses of HDRs in the foot (Positive BOLD) and hand (Negative BOLD) SMI across all subjects.

Negative activities under the RF condition were found in the left hand SMI (BA 3/4), SFG (BA 8), MeFG (BA 10), MFG (BA 11), inferior frontal gyrus (IFG) (BA 47), PHG (BA 36), fusiform gyrus (BA 37), anterior cingulate cortex (ACC) (BA 10, 24, and 32), and cingulate gyrus (CG) (BA 31). In the right hemisphere, deactivated regions were detected in the hand SMI (BA 3/4), SFG (BA 9), MeFG (BA 10), precentral gyrus (BA 6), IFG (BA 13), inferior temporal gyrus (BA 13), middle temporal gyrus (MTG) (BA 21), PHG (BA 36), ACC (BA 32), and CG (BA 31) (Fig 3, Table 1). The time courses of HDRs in the foot (Positive BOLD) and hand (Negative BOLD) SMI are shown in Fig 3C.

**Fig 4:**
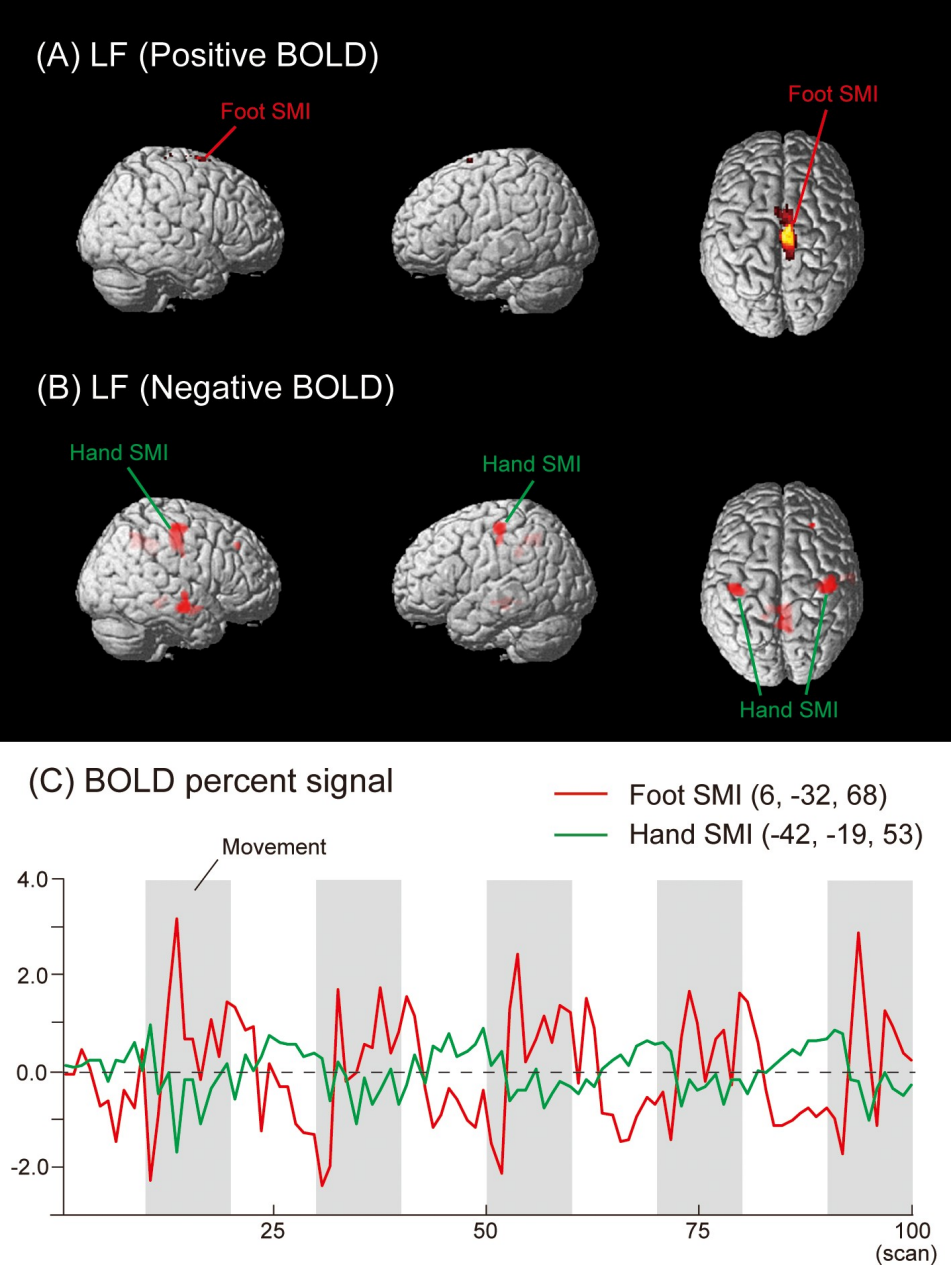
Group activation map showing (A) activated (Positive BOLD) and (B) deactivated (Negative BOLD) brain regions in the LF condition. (C) The mean time courses of HDRs in the foot (Positive BOLD) and hand (Negative BOLD) SMI across all subjects.

Negative activities under the LF condition were found in the left hand SMI (BA 3/4), precuneus (BA 7), PHG (BA 36), CG (BA 31), and lentiform nucleus. In the right hemisphere, deactivated regions were detected in the hand SMI (BA 3/4), MFG (BA 9), precentral gyrus (BA 6), precuneus (BA 7), STG (BA 22), MTG (BA 21), sub-gyral (BA 21), and PHG (Fig 4, Table 1). The time courses of HDRs in the foot (Positive BOLD) and hand (Negative BOLD) SMI are shown in Fig 4C.

### Comparison of BOLD strength between conditions in the ROI analysis

**Fig 5:**
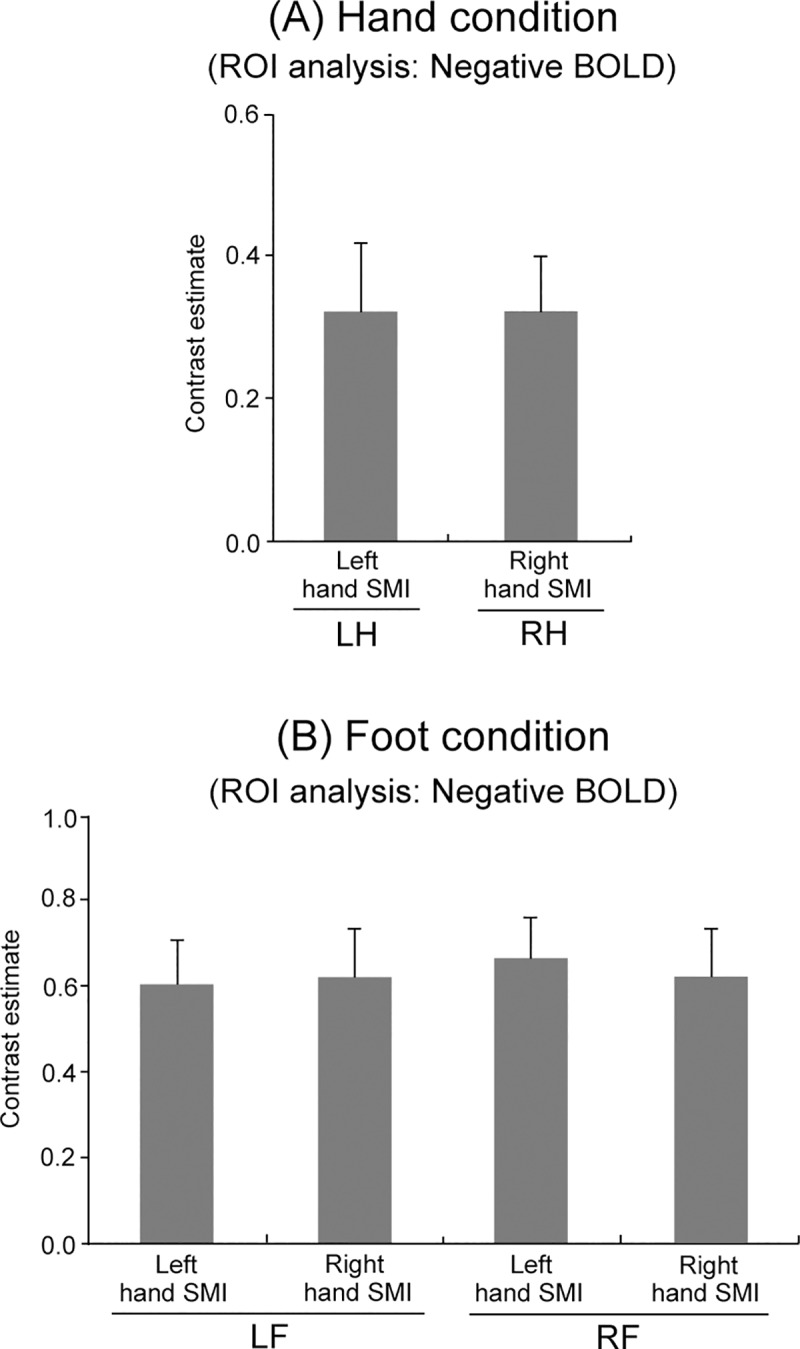
(A) ROI analysis for Negative BOLD responses in the ipsilateral hand SMI among Hand conditions. (B) ROI analysis for Negative BOLD responses in the bilateral hand SMI among Foot conditions.

In the ROI analysis, the ANOVA for the strength of the ipsilateral hand SMI of Negative BOLD during the right and left hand conditions revealed no significant main effect of Condition (Fig 5A). The ANOVA for the hand SMI strength of Negative BOLD during the right and left foot conditions also showed no significant main effect of Condition (Fig 5B).

### Conjunction analysis

**Fig 6:**
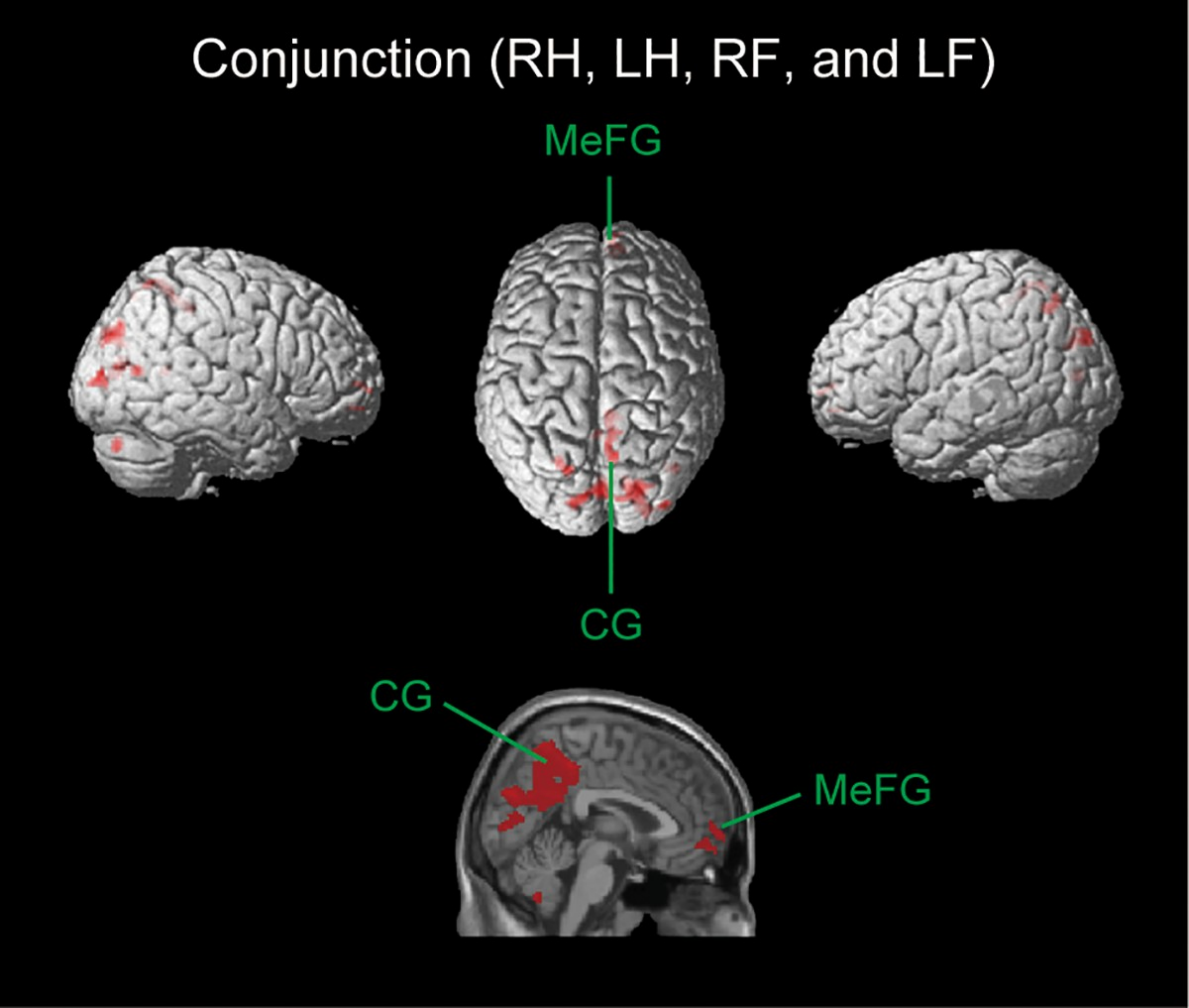
Brain regions commonly deactivated among RH, LH, RF, and LF conditions. Using the SPM template, areas showing a decrease in the BOLD signal are superimposed on a 3D-rendered standard brain (upper figures) as well as on the sagittal plane (lower figure). MeFG = medial frontal gyrus; CG = cingulate gyrus.

A conjunction analysis for common regions deactivated under all four (RH, LH, RF, and LF) conditions revealed significant deactivation in the left precuneus (BA 19), SPL (BA 7), cuneus (BA 17), and CG (BA 31), and in the right MeFG (BA 10), SFG (BA 10), precuneus (BA7), postcentral gyrus (BA 7), MTG (BA 39), cuneus (BA 7, 18 and 19), middle occipital gyrus (BA 18 and 19), posterior cingulate (BA 30), and cerebellum (declive) (Fig 6, Table 2).

**Table 2:** Deactivated regions for the conjunction analysis during RH, LH, RF, and LF conditions.

			Talairach coordinates			Z-score
Region	Side	BA	X	Y	Z	
Frontal Lobe						
Superior Frontal Gyrus	R	10	10	56	-11	3.59
Medial Frontal Gyrus	R	10	10	56	1	4.24
Parietal Lobe						
Precuneus	L	19	-8	-80	37	4.30
	R	7	6	-35	46	4.13
Postcentral Gyrus	R	7	8	-55	62	4.22
Superior Parietal Lobule	L	7	-24	-60	47	3.77
Temporal Lobe						
Middle Temporal Gyrus	R	39	42	-65	14	3.93
Occipital Lobe						
Cuneus	L	17	0	-79	11	3.75
	R	7	22	-78	33	4.58
	R	18	18	-77	17	5.23
	R	19	2	-80	32	4.56
Middle Occipital Gyrus	R	18	38	-85	13	4.10
	R	19	24	-85	10	4.44
Limbic Lobe						
Cingulate Gyrus	L	31	-2	-45	28	3.44
Posterior Cingulate	R	30	14	-50	14	3.47
Sub-lobar						
Cerebellum (Declive)	R		34	-79	-20	3.93

BA, Brodmann’s area; L, left hemisphere; R, right hemisphere

Activated regions in the Positive BOLD analysis are listed in S2 Table.

## Discussion

As described in the Introduction section, many studies have demonstrated deactivation in the ipsilateral MI during voluntary hand movements, suggesting transcallosal inhibition from one hemisphere to the other [1–8]. To the best of our knowledge, only one study has reported deactivation in brain regions other than the ipsilateral MI during actively initiated movements [2], and deactivation was observed in the precuneus when subjects performed a right-handed pinch grip. However, since only six right-handed subjects were used in that study, deactivation regions need to be confirmed using a larger number of subjects.

In the RH and LH conditions of the present study, Negative BOLD responses were detected not only in the ipsilateral SMI, but also in several brain regions, such as the MeFG, MFG, and SFG (Figs 1 and 2, and Table 1). These results suggest that Negative BOLD responses occurred during voluntary hand movements in addition to conventional transcallosal inhibition from the contralateral to ipsilateral hemisphere in the SMI. We also found Negative BOLD responses during the RF and LF conditions in the hand areas of the SMI, MeFG, SFG, IFG, MTG, PHG, ACC, CG, fusiform gyrus, and precuneus (Figs 3 and 4, and Table 1).

Negative BOLD responses were previously recorded during tongue movement [40]. Tongue movement generally showed a bilateral pattern of activation in the MI (a Positive BOLD) [41, 42]. Sakamoto and colleagues [40] did not detect a Negative BOLD response in cortical motor areas, such as the MI, SMA, premotor area (PM), and cerebellum, whereas this response was present in the paracentral lobule (BA 5), SPL, precuneus, and MTG. Sakamoto’s findings indicated that neural inhibition during voluntary movements occurred in brain regions not necessarily related to transcallosal inhibition mediated between the bilateral MI. On the other hand, as discussed in the Introduction section, Zeharia and colleagues [7] reported a Negative BOLD homunculus in the MI related to tongue, hand, and foot movements. They used a ROI analysis, which located a Negative BOLD homunculus in the MI, but not the SMA. Previous studies [2, 40] together with the present results indicate that a neural network of Negative BOLD signals exists during voluntary movements, which involves regions such as the precuneus, SPL, SFG, and MTG. Anatomical data demonstrated that the precuneus has strong reciprocal corticocortical connections with adjacent areas of the SPL, MTG, and prefrontal cortex (BA 8, 9, and 46) [43]. The prefrontal cortex, including the SFG, is also connected with many brain regions, such as the SPL, MTG, and limbic system, but not with primary sensory and motor cortices [44]. Negative BOLD signals in the MeFG and CG are discussed in more detail below.

We need to consider why bilateral hand SMI were deactivated under the RF and LF conditions (Figs 3 and 4). Since no direct anatomical connection was found between hand MI and foot MI [45], the functional hand-foot connection may be elicited by common inputs arising from secondary motor areas, such as the SMA and PM, rather than via horizontal connectivity within the MI [46]. If this is the case, the Negative BOLD responses of bilateral hand SMI during foot movements may be accomplished by inhibitory signals from the SMA and PM to the hand SMI. These signals may be generated together with excitatory signals to the foot SMI. Zeharia and colleagues [7] also suggested that Negative BOLD is related to the neural mechanisms underlying the balance between the suppression and activation of muscles across the body. Moreover, Volz and colleagues [47] used fMRI and dynamic causal modeling to investigate differences in the effective connectivity responsible for isolated movements of the hands or feet. This group showed that bilateral hand MI were inhibited during right or left foot movements. We assumed that the inhibition of bilateral hand SMI during unilateral foot movement was associated with adjusting body coordination in order to perform a specific, non-routine movement in the correct manner. This unilateral foot movement is in contrast to the more elementary, usual bi-pedal lower limb movements of our daily life and sport activities, such as walking, stepping, and running. It is interesting to note that some neuroimaging studies demonstrated that unilateral foot movement was generated with bilateral MI activity rather than with simple contralateral MI activity [47, 48]. Thus, unilateral plantar flexion and dorsiflexion in this study may be regarded as specific, non-routine movements for subjects. Specific brain activity may be required to perform the required movements correctly. To test this hypothesis, further studies are needed that utilize Positive and Negative BOLD signals to compare unilateral and bilateral lower limb movements.

In addition, Negative BOLD responses in the ipsilateral hand SMI did not significantly differ between the RH and LH conditions (Fig 5A), and Negative BOLD responses in the bilateral hand SMI also did not differ significantly between the RF and LF conditions (Fig 5B). These results suggest that the strength of the Negative BOLD response in the ipsilateral hand SMI during the hand condition and in the bilateral hand SMI during the foot condition may not be related to the laterality of handedness, irrespective of the right-handedness of all subjects. A number of studies using Positive BOLD responses noted that the recruitment of MI neurons during ipsilateral movement was more common in the left hemisphere (the left MI) than in the right hemisphere (the right MI) in right-handed subjects [5, 49, 50]. This finding indicates that asymmetric neural activity is involved in motor execution. To the best of our knowledge, differences between right and left hand or foot movement for Negative BOLD responses have not yet been examined in the whole brain. These results of this study indicate that there is no dominance in Negative BOLD responses in the ipsilateral hand SMI.

The conjunction analysis conducted in the present study demonstrated that a common Negative BOLD response occurred in the MeFG, CG, posterior cingulate, SPL, MTG, postcentral gyrus, precuneus, middle occipital gyrus, and cerebellum under all four conditions (RH, LH, RF, and LF) (Fig 6, and Table 2). It currently remains unclear why deactivation occur in these regions during voluntary movements of all body parts tested (right/left hand and right/left foot). Our hypothesis is that it occurred in ‘the context of a default mode network’. The default mode network has been identified in task-induced deactivation or in the brain activity associated with a passive fixation baseline condition relative to specific attention-demanding tasks [16, 51]. This network involves the MeFG (BA 9, 10, 24, and 32), medial and lateral parietal areas (BA 39 and 40), and posterior CG (BA 23, 29, 30, and 31) [17, 18, 52]. The crucial difference between previous studies investigating the default mode network and the present study is the task condition. Previous studies investigated neural activity during cognitive processing such as mind-wandering [53] and autobiographical remembering [54]. The present study did not use these cognitive tasks. However, the default mode network may be active not only during relatively unfocused cognitive tasks, but also during voluntary movements of the hands and feet.

Another possible explanation for the Negative BOLD responses observed in the present study involves the blood steal phenomenon. However, this explanation is not applicable to the present study because the regions that showed Negative and Positive BOLD responses were not adjacent (Figs 1–4). In future studies, diffusion tensor imaging may be useful for clarifying the neuropathology related to Negative and Positive BOLD responses in more detail.

A limitation of the present study was that the frequency of motor execution did not match between the hand and foot conditions (S1 Table). This occurred even though subjects were told to use a similar pace under all the conditions tested. We did not use a metronome during recording because other cognitive neural activities, such as auditory processing and audio-motor matching activity, may be included in Negative and Positive BOLD signals. We speculated that the motor execution of repetitive plantar flexion and dorsiflexion with the right or left ankle may be more difficult to perform than that of repetitive extension and flexion of the right or left hand. Positive BOLD signals in the SMI were enhanced as the frequency of motor execution increased [5, 55]. In Sadato’s study [55], movements of the right index finger were paced with the steady beat (0.25, 0.5, 0.75, 1, 2, 2.5, 3, and 4 Hz) of a metronome, which was placed close to the subject’s ear. They classified these paces into four categories: very slow (0.25 and 0.5 Hz), slow (0.75 and 1 Hz), fast (2 and 2.5 Hz), and very fast (3 and 4 Hz). They then compared the magnitude of brain activity for the different rates. The magnitude in the SMI was almost the same between 0.75 and 1 Hz at the slow rate. In the present study, the mean values of frequencies across all subjects were 1.33 Hz in the RH condition, 1.33 Hz in the LH condition, 1.16 Hz in the RF condition, and 1.14 Hz in the LF condition. The difference between the right hand and foot was approximately 0.17 Hz, and approximately 0.19 Hz between the left hand and foot. Therefore, although the difference in frequency was significant, the effects of frequency on Negative BOLD signals between the hand and foot conditions appeared to be negligible.

In addition, no Negative BOLD responses under the LH condition were detected using the FWE with a cluster-level threshold of p < 0.05, whereas those for SMI under the RH, RF, and LF conditions were found (Table 1). These findings suggest differences in the strength of Negative BOLD responses among conditions. The detailed mechanisms should be clarified in future studies.

## Conclusion

Negative BOLD responses during hand movements were observed not only in the ipsilateral hemisphere of SMI, but also in other brain regions, such as the MeFG, MFG, and SFG. Furthermore, Negative BOLD responses during foot movement were detected in the bilateral hand SMI as well as in the MeFG, SFG, IFG, MTG, PHG, ACC, CG, fusiform gyrus, and precuneus. There were also two common deactivated regions, the MeFG and CG, which were independent of the movements of the upper and lower limbs. These regions closely corresponded to the default mode network, which was previously reported.

We suggest that the Negative BOLD responses observed during voluntary movements are produced by three mechanisms: (1) transcallosal inhibition from the contralateral to ipsilateral hemisphere in the SMI, (2) the deactivated neural network with several brain regions, and (3) the default mode network in the MeFG and CG. The present results extend the potential of utilizing Negative and Positive BOLD signal comparisons to obtain a better understanding of the motor control system.

## Supporting information

S1 Table The number of hand and foot movements for each subject under each condition. (DOCX)

S2 Table Activation regions under each condition. (DOCX)
