# Supplementary material for: Negative BOLD responses during hand and foot movements: An fMRI study
Source: PLoS One. 2019 Apr 19;14(4):e0215736. doi: 10.1371/journal.pone.0215736 (PMC6474656; doi:10.1371/journal.pone.0215736)
Supplement: S1 Table — (DOCX) [file pone.0215736.s001.docx]

**Supplementary Table S1:** The number of hand and foot movements for each subject under each condition

| Subject | 1st | 2nd | 3rd | 4th | 5th | Ave |
| --- | --- | --- | --- | --- | --- | --- |
| RH condition |  |  |  |  |  |  |
| A | 58 | 56 | 60 | 59 | 61 | 58.8 |
| B | 59 | 62 | 60 | 59 | 58 | 59.6 |
| C | 36 | 35 | 35 | 37 | 37 | 36.0 |
| D | 28 | 28 | 28 | 29 | 28 | 28.2 |
| E | 33 | 32 | 32 | 32 | 33 | 32.4 |
| F | 34 | 35 | 36 | 35 | 35 | 35.0 |
| G | 45 | 44 | 41 | 40 | 38 | 41.6 |
| H | 43 | 45 | 47 | 48 | 49 | 46.4 |
| I | 40 | 36 | 37 | 36 | 34 | 36.6 |
| J | 20 | 22 | 24 | 24 | 24 | 22.8 |
| K | 48 | 45 | 48 | 48 | 49 | 47.6 |
| L | 69 | 54 | 52 | 67 | 70 | 62.4 |
| M | 22 | 22 | 21 | 21 | 19 | 21.0 |
| N | 40 | 40 | 39 | 40 | 41 | 40.0 |
| O | 31 | 29 | 38 | 28 | 28 | 30.8 |
| Ave | 40.4 | 39.0 | 39.9 | 40.2 | 40.3 | 39.9 |
|  |  |  |  |  |  |  |
| LH condition |  |  |  |  |  |  |
| A | 58 | 66 | 66 | 67 | 60 | 63.4 |
| B | 59 | 59 | 61 | 62 | 59 | 60.0 |
| C | 37 | 37 | 33 | 37 | 36 | 36.0 |
| D | 30 | 29 | 30 | 29 | 30 | 29.6 |
| E | 33 | 32 | 32 | 33 | 31 | 32.2 |
| F | 33 | 35 | 36 | 37 | 35 | 35.2 |
| G | 38 | 36 | 35 | 35 | 36 | 36.0 |
| H | 43 | 44 | 43 | 45 | 45 | 44.0 |
| I | 34 | 35 | 36 | 36 | 34 | 35.0 |
| J | 31 | 28 | 28 | 28 | 26 | 28.2 |
| K | 44 | 43 | 43 | 44 | 43 | 43.4 |
| L | 64 | 70 | 78 | 76 | 76 | 72.8 |
| M | 31 | 30 | 28 | 26 | 24 | 27.8 |
| N | 29 | 31 | 30 | 30 | 32 | 30.4 |
| O | 28 | 26 | 27 | 27 | 26 | 26.8 |
| Ave | 39.5 | 40.1 | 40.4 | 40.8 | 39.5 | 40.1 |
|  |  |  |  |  |  |  |
| RF condition |  |  |  |  |  |  |
| A | 58 | 57 | 61 | 63 | 63 | 60.4 |
| B | 59 | 59 | 60 | 59 | 60 | 59.4 |
| C | 37 | 39 | 39 | 36 | 36 | 37.4 |
| D | 29 | 27 | 27 | 27 | 26 | 27.2 |
| E | 40 | 35 | 34 | 32 | 33 | 34.8 |
| F | 33 | 31 | 32 | 32 | 32 | 32.0 |
| G | 39 | 35 | 34 | 31 | 32 | 34.2 |
| H | 37 | 38 | 41 | 42 | 43 | 40.2 |
| I | 35 | 34 | 36 | 34 | 33 | 34.4 |
| J | 21 | 20 | 21 | 21 | 20 | 20.6 |
| K | 32 | 34 | 36 | 35 | 34 | 34.2 |
| L | 38 | 34 | 33 | 31 | 32 | 33.6 |
| M | 14 | 15 | 14 | 14 | 14 | 14.2 |
| N | 32 | 32 | 30 | 30 | 31 | 31.0 |
| O | 29 | 29 | 28 | 29 | 29 | 28.8 |
| Ave | 35.5 | 34.6 | 35.1 | 34.4 | 34.5 | 34.8 |
|  |  |  |  |  |  |  |
| LF condition |  |  |  |  |  |  |
| A | 55 | 56 | 59 | 60 | 61 | 58.2 |
| B | 56 | 54 | 55 | 55 | 58 | 55.6 |
| C | 34 | 34 | 36 | 34 | 36 | 34.8 |
| D | 36 | 34 | 33 | 31 | 30 | 32.8 |
| E | 32 | 31 | 32 | 32 | 31 | 31.6 |
| F | 34 | 34 | 33 | 32 | 32 | 33.0 |
| G | 33 | 30 | 28 | 28 | 27 | 29.2 |
| H | 37 | 36 | 37 | 37 | 36 | 36.6 |
| I | 34 | 36 | 40 | 36 | 34 | 36.0 |
| J | 22 | 22 | 22 | 21 | 22 | 21.8 |
| K | 37 | 35 | 34 | 35 | 35 | 35.2 |
| L | 35 | 34 | 32 | 35 | 37 | 34.6 |
| M | 16 | 15 | 14 | 14 | 14 | 14.6 |
| N | 31 | 32 | 30 | 30 | 32 | 31.0 |
| O | 28 | 28 | 29 | 29 | 29 | 28.6 |
| Ave | 34.7 | 34.1 | 34.3 | 33.9 | 34.3 | 34.2 |

There was a significant main effect of limb (hand vs. foot).
