# Supplementary material for: Negative BOLD responses during hand and foot movements: An fMRI study
Source: PLoS One. 2019 Apr 19;14(4):e0215736. doi: 10.1371/journal.pone.0215736 (PMC6474656; doi:10.1371/journal.pone.0215736)
Supplement: S2 Table — (DOCX) [file pone.0215736.s002.docx]

**Supplementary Table S2:** Activation regions under each condition

|  |  |  | Talairach coordinates | | | Z-score |
| --- | --- | --- | --- | --- | --- | --- |
| Region | Side | BA | X | Y | Z |  |
| <RH condition> |  |  |  |  |  |  |
| Frontal Lobe |  |  |  |  |  |  |
| Superior Frontal Gyrus | R | 6 | 4 | 10 | 53 | 4.46 |
| Medial Frontal Gyrus | L | 6 | -10 | -1 | 52 | 5.52 |
|  | R | 6 | 10 | 5 | 51 | 3.78 |
| Middle Frontal Gyrus | L | 6 | -38 | -3 | 57 | 3.73 |
| Inferior Frontal Gyrus | R | 44 | 50 | 14 | 10 | 3.88 |
|  | R | 47 | 53 | 17 | -4 | 4.80 |
| Precentral Gyrus | L | 4 | -36 | -17 | 51 | 5.52 |
|  | L | 6 | -36 | -18 | 64 | 5.03 |
|  | L | 44 | -46 | -2 | 7 | 4.95 |
|  | R | 44 | 55 | 10 | 7 | 3.82 |
| Cingulate Gyrus | L | 32 | -8 | 9 | 33 | 4.78 |
| Paracentral Lobule | L | 31 | -8 | -32 | 43 | 4.71 |
| Parietal Lobe |  |  |  |  |  |  |
| Postcentral Gyrus | L | 3 | -40 | -28 | 50 | 4.16 |
| Supramarginal Gyrus | L | 40 | -55 | -37 | 28 | 5.32 |
| Temporal Lobe |  |  |  |  |  |  |
| Superior Temporal Gyrus | L | 22 | -51 | 2 | 4 | 5.10 |
|  | R | 22 | 51 | 10 | 0 | 4.34 |
| Limbic Lobe |  |  |  |  |  |  |
| Cingulate Gyrus | L | 24 | -8 | -3 | 48 | 5.91 |
| Sub-lobar |  |  |  |  |  |  |
| Insula | L | 13 | -44 | -26 | 18 | 5.10 |
| Lentiform Nucleus | L |  | -18 | -7 | 6 | 4.02 |
| Thalamus | L |  | -16 | -19 | 10 | 4.42 |
| Cerebellum (Culmen) | R |  | 6 | -55 | -4 | 5.33 |
|  |  |  |  |  |  |  |
| <LH condition> |  |  |  |  |  |  |
| Frontal Lobe |  |  |  |  |  |  |
| Superior Frontal Gyrus | L | 6 | -8 | 7 | 55 | 4.29 |
|  | L | 10 | -30 | 51 | 20 | 3.98 |
|  | R | 6 | 6 | 9 | 55 | 4.79 |
|  | R | 10 | 32 | 55 | 17 | 4.47 |
| Middle Frontal Gyrus | L | 10 | -38 | 49 | 14 | 4.35 |
|  | R | 6 | 20 | 20 | 56 | 4.58 |
| Inferior Frontal Gyrus | L | 9 | -59 | 9 | 24 | 4.20 |
| Precentral Gyrus | R | 4 | 38 | -13 | 49 | 5.65 |
|  | R | 6 | 28 | -10 | 63 | 4.91 |
|  | R | 44 | 51 | 12 | 10 | 4.75 |
| Cingulate Gyrus | R | 32 | 2 | 19 | 34 | 4.92 |
| Parietal Lobe |  |  |  |  |  |  |
| Inferior Parietal Lobule | L | 40 | -55 | -36 | 24 | 4.05 |
|  | R | 40 | 55 | -30 | 31 | 4.04 |
| Postcentral Gyrus | R | 3 | 42 | -20 | 56 | 4.31 |
| Temporal Lobe |  |  |  |  |  |  |
| Superior Temporal Gyrus | L | 22 | -55 | 6 | -2 | 4.15 |
|  | R | 22 | 51 | 10 | 0 | 4.25 |
| Limbic Lobe |  |  |  |  |  |  |
| Cingulate Gyrus | L | 24 | -10 | 13 | 31 | 4.45 |
|  | R | 24 | 12 | 2 | 46 | 4.79 |
|  | R | 31 | 10 | -9 | 47 | 3.97 |
| Anterior Cingulate | R | 24 | 6 | 24 | 19 | 4.03 |
| Sub-lobar |  |  |  |  |  |  |
| Insula | L | 13 | -36 | 14 | 9 | 4.49 |
|  | R | 13 | 38 | 10 | -4 | 5.25 |
| Caudate | R |  | 10 | 5 | 13 | 5.63 |
| Claustrum | R |  | 34 | 12 | 7 | 4.61 |
| Lentiform Nucleus | L |  | -20 | 6 | 13 | 4.32 |
|  | R |  | 16 | -2 | -5 | 4.69 |
| Thalamus | L |  | -6 | -23 | 3 | 4.06 |
|  | R |  | 16 | -13 | 4 | 5.48 |
| Cerebellum (Culmen) | L |  | -6 | -59 | -5 | 4.76 |
| Cerebellum (Declive) | L |  | -8 | -63 | -14 | 4.76 |
| Cerebellum (Dentate) | L |  | -14 | -59 | -19 | 5.25 |
| Brainstem | R |  | 10 | -16 | -9 | 5.20 |
|  |  |  |  |  |  |  |
| <RF condition> |  |  |  |  |  |  |
| Frontal Lobe |  |  |  |  |  |  |
| Superior Frontal Gyrus | L | 4/6 | -4 | -6 | 65 | 5.76 |
|  | R | 6 | 2 | 7 | 53 | 4.02 |
| Medial Frontal Gyrus | L | 6 | -8 | -20 | 56 | 6.89 |
| Precentral Gyrus | L | 44 | -50 | 0 | 6 | 4.39 |
| Cingulate Gyrus | L | 32 | -8 | 9 | 33 | 4.65 |
| Temporal Lobe |  |  |  |  |  |  |
| Superior Temporal Gyrus | L | 41 | -46 | -28 | 18 | 5.44 |
|  | R | 22 | 48 | 6 | -2 | 4.38 |
| Parietal Lobe |  |  |  |  |  |  |
| Postcentral Gyrus | R | 40 | 59 | -26 | 22 | 4.13 |
| Limbic Lobe |  |  |  |  |  |  |
| Cingulate Gyrus | L | 24 | -6 | 0 | 42 | 4.36 |
| Sub-lobar |  |  |  |  |  |  |
| Insula | L | 13 | -53 | -32 | 18 | 5.54 |
| Thalamus | L |  | -22 | -19 | 12 | 4.01 |
| Claustrum | L |  | -32 | 2 | 7 | 4.31 |
| Cerebellum (Culmen) | R |  | 2 | -49 | -6 | 4.68 |
|  |  |  |  |  |  |  |
| <LF condition> |  |  |  |  |  |  |
| Frontal Lobe |  |  |  |  |  |  |
| Superior Frontal Gyrus | L | 6 | -6 | 7 | 66 | 4.12 |
|  | R | 6 | 2 | 1 | 66 | 4.47 |
| Medial Frontal Gyrus | L | 6 | 0 | -16 | 63 | 5.25 |
|  | R | 4/6 | 6 | -13 | 58 | 4.81 |
| Paracentral Lobule | R | 6 | 6 | -32 | 68 | 4.15 |
| Limbic Lobe |  |  |  |  |  |  |
| Cingulate Gyrus | R | 24 | 4 | -4 | 44 | 4.25 |
| Sub-lobar |  |  |  |  |  |  |
| Cerebellum (Culmen) | L |  | -10 | -44 | -16 | 4.64 |

BA, Brodmann’s area; L, left hemisphere; R, right hemisphere
